# Methylene blue adsorption mechanism of activated carbon synthesised from cashew nut shells†

**DOI:** 10.1039/d1ra04672a

**Published:** 2021-08-03

**Authors:** Nguyen Hoc Thang, Dinh Sy Khang, Tran Duy Hai, Dinh Thi Nga, Phan Dinh Tuan

**Affiliations:** Department of Materials Technology, Faculty of Chemical Technology, Ho Chi Minh City University of Food Industry 140 Le Trong Tan Street, Tan Phu District Ho Chi Minh City Vietnam; Faculty of Environment, Ho Chi Minh City University of Natural Resources and Environment 236B Le Van Sy Street, Tan Binh District Ho Chi Minh City Vietnam khangds@hcmunre.edu.vn; Research Institute for Sustainable Development, Ho Chi Minh City University of Natural Resources and Environment 236B Le Van Sy Street, Tan Binh District Ho Chi Minh City Vietnam

## Abstract

Activated carbon produced from agricultural products and wastes has been applied widely to remove pollutants in the field of waste water treatment. However, the cost of this adsorbent depends so much on the raw material sources. Therefore, the approach of producing activated carbon from agricultural waste is strongly recommended due to economic advantages and environmental protection. One of the potential feed-stocks for the activated carbon production is cashew nut shell (CNS) waste which could reduce the negative impacts to the environment from the cashew nut processing industry and simultaneously enhance the values of the related products. This study focused on evaluating the influences of variable factors, such as activation temperature and time, on the properties of the activated carbon obtained from CNS. Methylene blue (MB) adsorption was applied to understand the adsorption mechanism of the products. The results show that increasing the activation temperature led to a rise in the adsorption capacity of the activated carbon within the temperature range of 800 to 850 °C. Otherwise, the values were reduced when the temperature was greater than 850 °C and this was related to the thermal decomposition of carbon. The adsorption capacity also increased when the activation time was changed from 30 min to 50 min. However, in the activation time range from 50 to 70 min, there was a reduction of the adsorption capacity of CNS-based activated carbon. The results also show that the MB adsorption of the activated carbon occurred with one-site-occupancy in the first layer and then layer-by-layer adsorption formation.

## Abstract 2

Activated carbon produced from agricultural products and wastes has been applied widely to remove pollutants in the field of waste water treatment.

## Graphical abstract

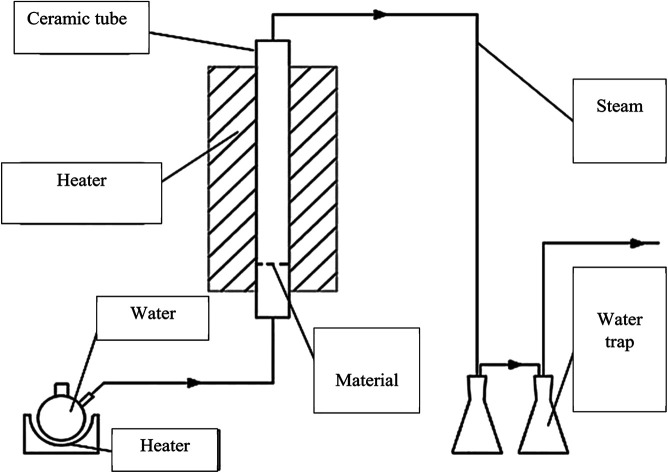

## Introduction

Vietnam is one of the biggest producers of cashew nuts, producing approximately 300 thousand tons of cashew nuts per year, accounting for up to 28% of the total world production. Vietnam is also one of the top ten countries for growing cashew nuts and has promoted significantly the capacity of cashew nut production in this country.^1^ However, this also releases a huge amount of cashew nut shell (CNS) waste into the environment. The CNS has been considered as a hazardous solid waste that should be managed properly. These solid waste resources are currently causing many issues such as negative impacts on the environment, they are easily ignited and so can cause fires in the dry conditions of tropical weather as in Vietnam, large areas are used to contain the CNS waste and this increases the cost of cashew nut production, and so on. In addition, diversifying the sources of activated carbon production for environmental treatment processes should always be promoted. Thus, utilisation of the CNS waste as a raw material for activated carbon production should be considered as a new approach in solid waste treatment based on the current conditions in Vietnam. This will also enhance the value of cashew nut-based products as well as reducing the environmental impacts from this industry.

The most common commercial adsorbent for waste water treatment is activated carbon, however, it has the disadvantage of a high cost because of the production processes.^2^ Activated carbon has been widely recommended as highly effective adsorbent in waste water treatment due to its large surface area, high degree of surface reactivity, and abundant availability of material.^3,4^ It is applied in the pollutant absorption of both organic and inorganic compounds.^5^ There are many sources of materials from agricultural products and wastes such as bamboo, wood, coconut shells, and rice husks which were used to produce activated carbon. However, the production costs are still higher than that of other adsorbents.^5,6^ Previous studies showed that the activated carbon could be produced from the low-cost raw materials. Moreover, the properties of the products are significantly efficient in adsorbing pollutants.^7^ However, it is necessary for comprehensive investigations to be done on the CNS-based activated carbon and its absorption mechanism. Therefore, this study aims to establish a new activated carbon source from CNS waste that is the hazardous waste source of cashew nut production in Vietnam.

The properties of activated carbon produced from CNS waste could be improved to increase the capacity of the pollutants' absorption in processes of waste water treatment. The pollutants' absorption capacity of this activated carbon source is affected by activation conditions such as temperature and time as well as absorption conditions, which were all investigated simultaneously in this study. A kinetic study was useful to determine how significantly these parameters influence the absorption efficiency of CNS-based activated carbon. This study also evaluated, and explained, the adsorption mechanism of the pollutants absorbed onto the CNS-based activated carbon. These models are useful to control the system operation and optimise the adsorption capacity. The most well-known isotherm adsorption models are Langmuir,^8^ Freundlich,^9^ Dubinin–Radushkevich,^10^ n-layer BET,^11^ Guggenheim–Anderson–de-Boer.^12^ There are some kinetic models of adsorption processes such as Weber and Morris,^13^ pseudo-first-order and pseudo-second-order,^14^ Elovic^15^ and others.^16,17^ There are several operational parameters such as pH and initial concentration of pollutant solution which were considered to establish the adsorption models in this study.

## Materials and methods

### Activated carbon preparation

**Fig. 1:**
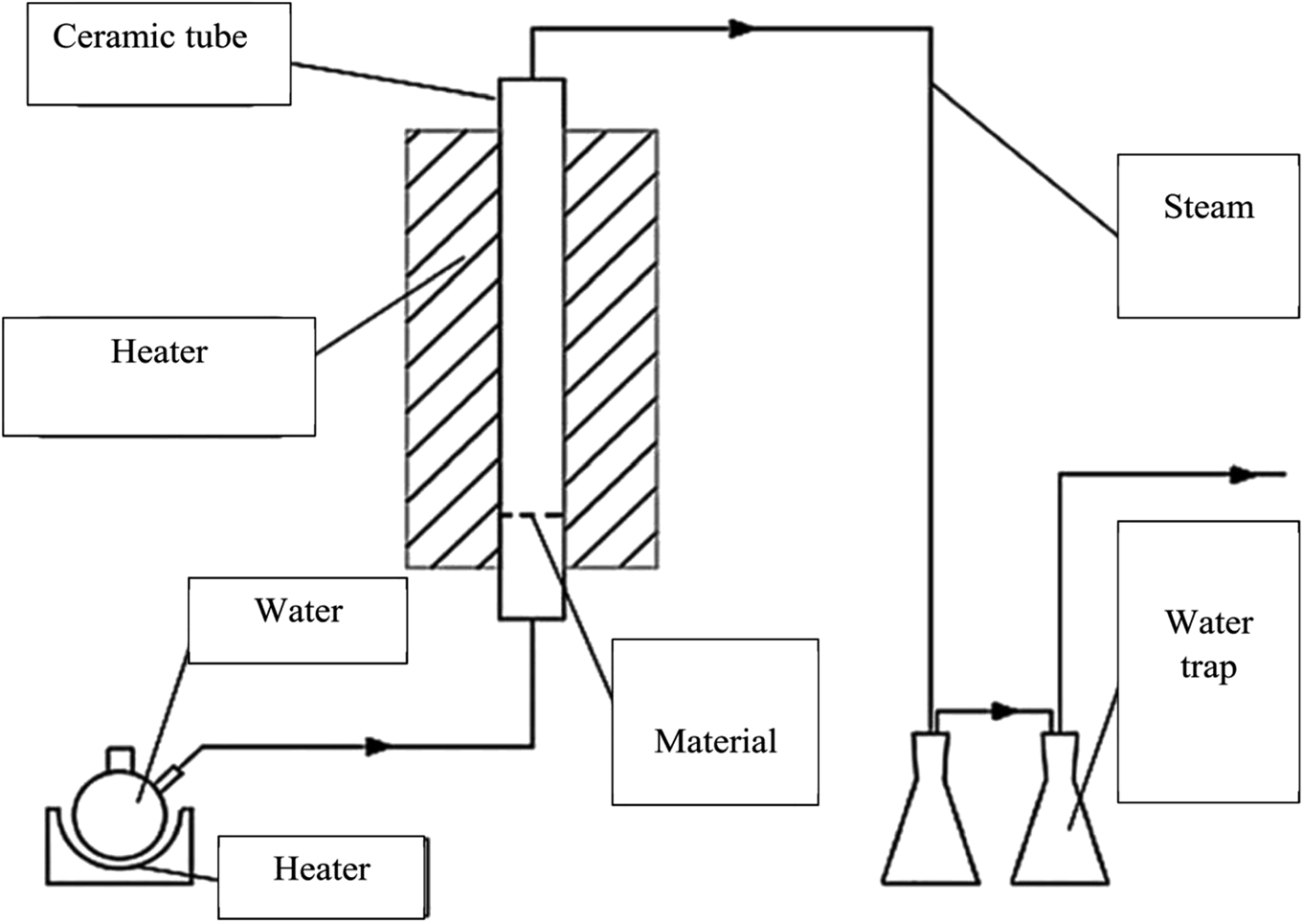
The production system for CNS-based activated carbon.

The CNS waste was collected from the biggest cashew nut factory in Binh Phuoc province, Vietnam. The raw material was dried, ground and sieved in the size range of 0.1 to 4.0 mm (mesh no. 5 to 140). The experimental system was established as shown in Fig. 1. A fixed-bed vertical tubular reactor with an inside diameter of 6 cm was used to contain the samples. The CNS coarse particles were pyrolyzed at 500 °C for 1 h and this was assisted by a high temperature updraft stream of N_2_ with a velocity of 1.4 mL min^−1^. Then the reaction temperature was increased to reach 800, 850, and 900 °C. The nitrogen flow was switched to a normal pressure stream with a velocity of 0.5 mL min^−1^ during the reaction time. The activated carbon product was then ground and sieved to reach particle sizes from 0.1 to 1.0 mm using the mesh no. 20 to 140.

### Analysis of the CNS-based activated carbon

The specific surface area (*S*_BET_), pore size distribution and pore volume (*V*_pore_) of the CNS-based activated carbon were determined using the nitrogen adsorption isotherm with a Nova 2200e® (Quantachrome Instruments, USA) according to the BET theory, the BJH method and density functional theory, respectively. The samples were also characterised for vibrations of the chemical functional groups using Fourier transform infrared spectroscopy (FT-IR) with the wavenumber in the range of 400 to 4000 cm^−1^. The FT-IR analyses were carried out on 8400S spectrophotometer (Shimadzu, Japan). OriginPro 2017 software (OriginLab, USA) was used for non-linear fitting between the experimental data and the predicted model. The differential equations were numerically solved using the Runge–Kutta 4th order method.

### Adsorption performance

Methylene blue (MB) from the Merck Group was used as a model organic pollutant to investigate the adsorption models. The stock solution (1000 mg L^−1^) was prepared by dissolving the 1000 mg of MB in 1.0 L of distilled water. The concentrations of MB prepared were 100, 150, 200, 250 and 300 mg L^−1^. The pH parameters of the MB solutions were adjusted to expected values of 2, 4, 6, 8, and 10 with 0.010 M of NaOH and/or HCl solutions. The container containing the mixture of adsorbent particles and adsorbate molecules was placed in a temperature-controlled shaker bath at a temperature of 27 °C. The MB adsorption capacity *q*_t_ was calculated from the remaining MB concentration by the following equation:1
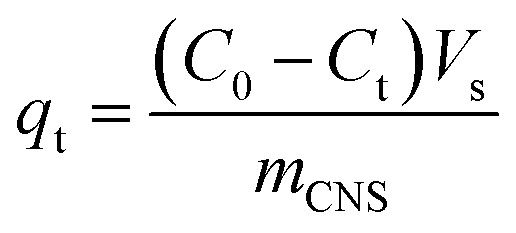
where, *V*_s_ is the volume of the solution (0.25 L), and *m*_CNS_ is the amount of CNS-based activated carbon (0.25 mg) used.

When the adsorption tends to equilibrium, the adsorption capacity and the remaining MB concentration are indicated by *q*_e_ and *C*_e_, respectively.

## Results and discussion

### Effects of activation temperature and time on the characterisation of the CNS-based activated carbons

The effects of activation temperature on the conversion efficiency of the activated carbon production are shown in Table 1.

**Table 1:** Effect of activation temperature on conversion efficiency

Temperature °C	Activation efficiency %	Total efficiency %
800	78.7	22.0
850	64.0	17.9
900	58.0	16.2

**Fig. 2:**
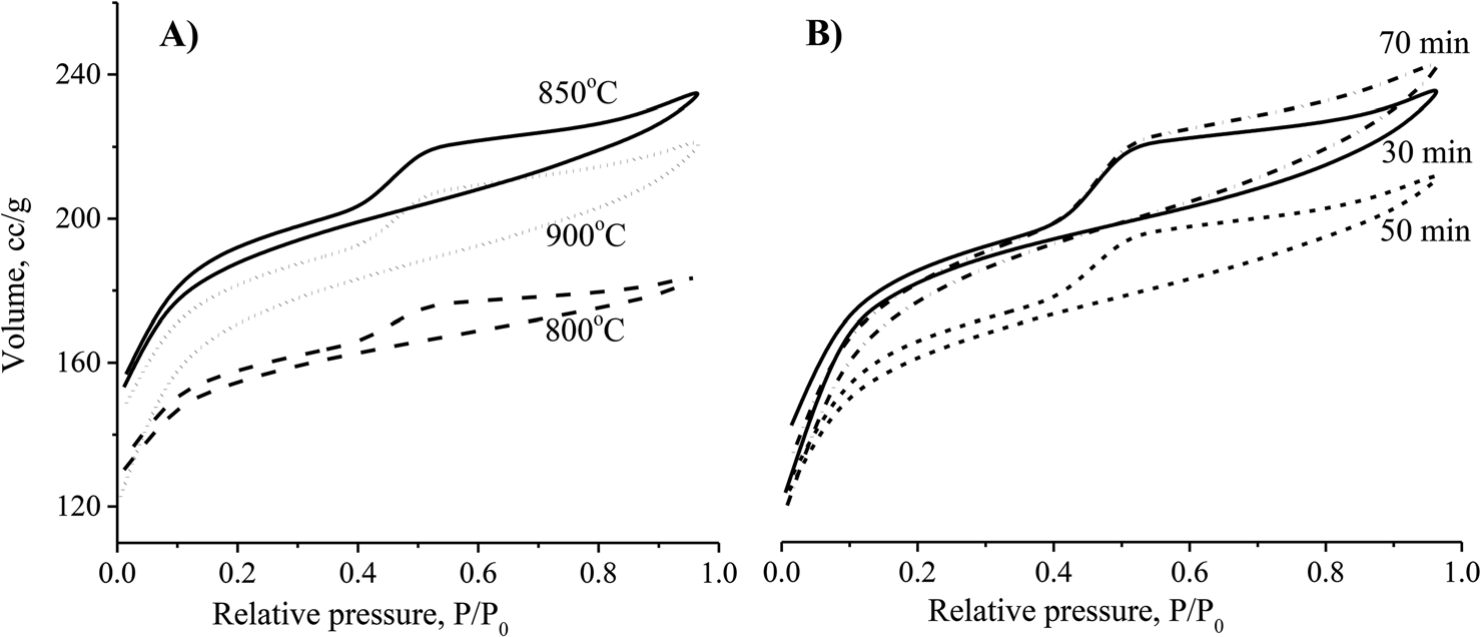
The effects of activation temperature (A) and activation time (B) on the N_2_ adsorption/desorption capability of the CNS-based activated carbons.

The results showed that an increase of the activation temperature reduced the conversion efficiency of the activated carbon production. This was the result of a reaction between the molecule of H_2_O and carbon at a high temperature. The results also indicated that the specific surface area of the CNS-based activated carbon material increased from 518.9 m^2^ g^−1^ to 678.8 m^2^ g^−1^ and the pore volume increased from 0.271 cm^3^ g^−1^ to 0.342 cm^3^ g^−1^ when the activation temperature was changed from 800 °C to 850 °C. Thus, the adsorption capacity of activated carbon in Fig. 2 increased with the increase of the activation temperature. However, the adsorption capacity decreased with an activation temperature higher than 850 °C. At high temperatures, the steam distended the pore shapes of the carbon structures. Therefore, the optimised temperature must be considered for both production efficiency and specific surface area of the CNS-based activated carbon material.

**Fig. 3:**
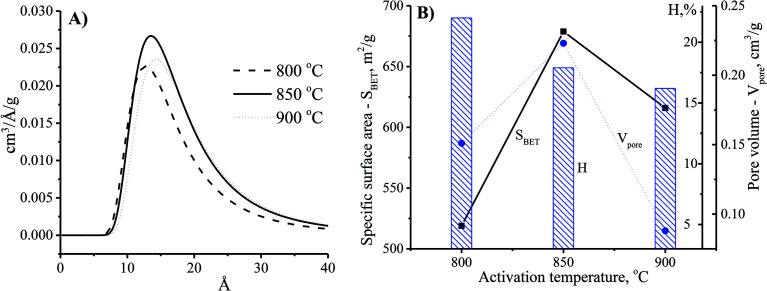
The effects of activation temperature on the pore size distribution (A) and physio-chemical properties (B) of the CNS-based activated carbon material with the activation time at 30 min.

Theoretically, the adsorption capacity of activated carbon significantly depends on its pore diameter in structure of carbon-based material related to its pore volume. In Fig. 3A, the pore size of the CNS-based activated carbons are from 10 to 20 Å. These results show that there was limited efficiency of MB adsorption due to the large size of dye molecule from 5.91 to 13.82 Å. The pore sizes of the CNS-based activated carbon were increased according to the increase of activation temperature. Consequently, the average mesoporous diameters of the pores in the materials were 12.8, 13.6 and 14.2 Å for the activation temperatures of 800, 850, and 900 °C, respectively. The reactions of the volatile matter and the activated agents were stronger at higher temperatures resulting in not only formation of new pores but also enhancement of the pore diameter.^18^ Therefore, the mesoporous volume (*V*_pore_) and surface area (*S*_BET_) of the CNS carbon activated at 850 °C were higher than that activated at 800 °C as shown in the Fig. 3B. However, both the *V*_pore_ and *S*_BET_ of the CNS-based activated carbon were decreased when the activation temperature reached 900 °C. This was related to the extension of the pores, sintering, and diffusion of fine particles.^19^ The variations of the *V*_pore_ and *S*_BET_ with the activation temperature were also similar to these. Fig. 3B also shows a decrease of the processing efficiency (H, %) indicated by the mass percentage of product per initial material.

**Fig. 4:**
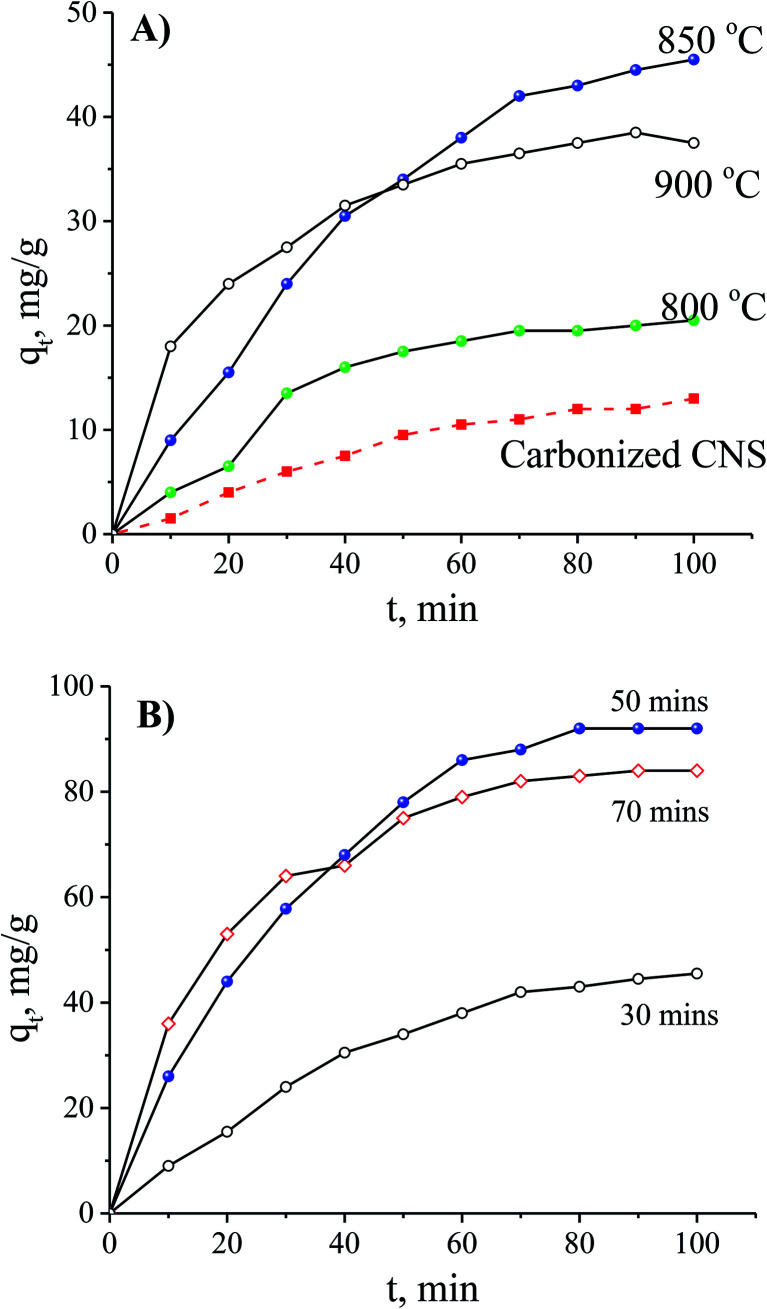
The effects of the activation temperature (A) and the activation time (B) on MB adsorption with an initial concentration of 100 mg L^−1^.

In order to optimise the activation temperature, many experiments were conducted for MB adsorption on the CNS-based activated carbons. The adsorption capacities at different activation time are shown in Fig. 4. The results showed that the CNS pyrolyzed carbon was very poor for MB removal with a low MB adsorption capacity. The highest value was 13 mg g^−1^ within 100 min resulting which was unfeasible for use in an application for an adsorbent. In the activation stage, the volatile matter was thermo-chemically decomposed and the active surface was exposed.^18^ These results show a good agreement between MB adsorption capacity and the positively changes of *V*_pore_, *S*_BET_ values. The required time for the equilibrium was about 120 min for all of the adsorbents tested.

**Fig. 5:**
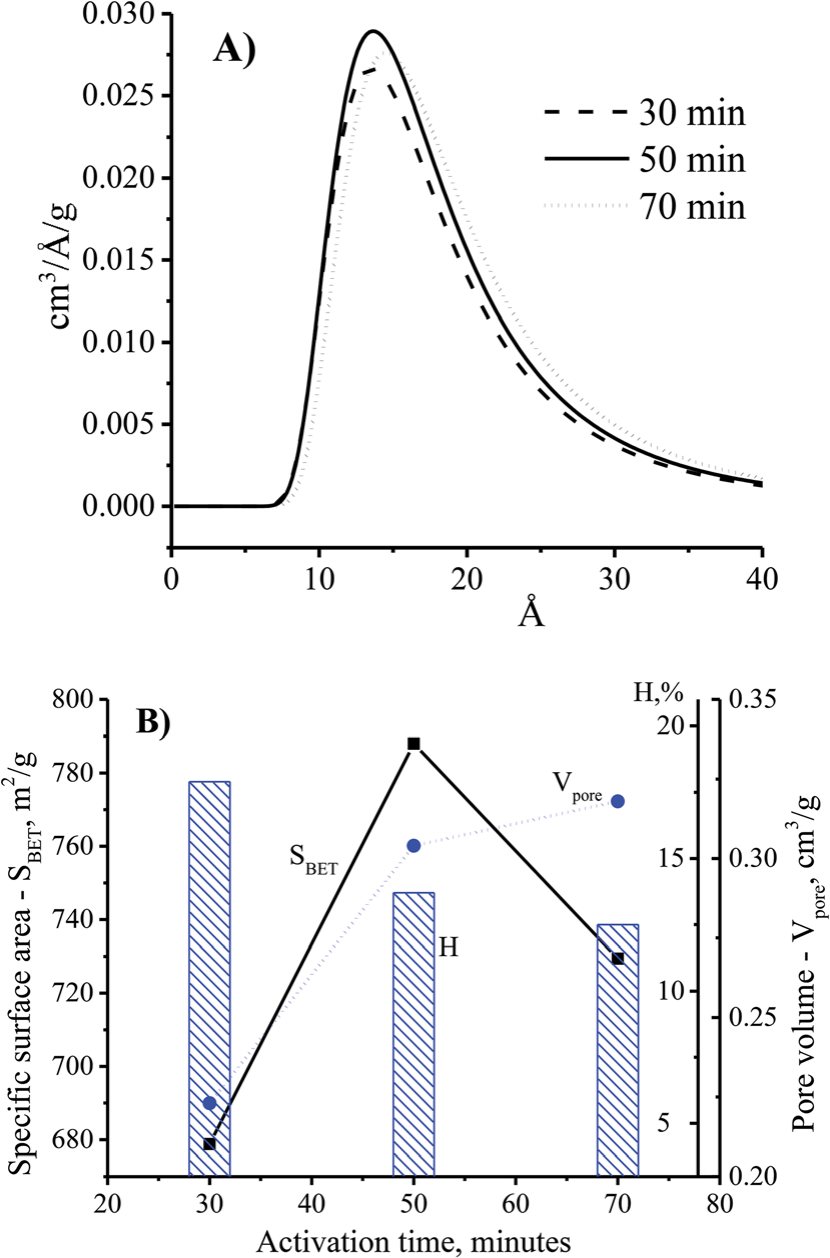
The effects of activation time on pore size distribution (A) and physiochemical properties (B) of the CNS-based activated carbon at the activation temperature of 850 °C.

The adsorption ability of activated carbon depended on the pore size distribution which was related to the adsorbate trapping and the capability of the adsorbent to retain the adsorbate.^18^Fig. 5A shows the enhancement of the pore diameter of the CNS-based activated carbon with the increase of the activation time. In comparison with the CNS carbon activated within 30 min, the pore size of the CNS carbon activated within 50 min was unchanged as shown in Fig. 5A. However, the pore volume of the activated carbon could be improved by increasing the surface area as shown in Fig. 5B. This is explained by the fact that the activation process occurred on the surface of the carbon particles, and the CNS-based activated carbon core was ignored. Thus, the surface layers of the carbon structure influenced the pore structure of the CNS-based activated carbons. When the activation time was increased to 70 min, the material had not only been completely converted into ash as shown in Fig. 4B, but there was also a slight increase of the diameter and the volume of the pores as shown in Fig. 5B, due to the melting and sintering of micropores into mesopores.^20^ This caused a partial collapse of the microporous structures resulting in a lower surface area of the CNS carbon activated for 70 min when compared with that of 50 min. The longer the activation time is, the more the processing efficiency was reduced due to the removal of volatiles as shown in Fig. 5B. This was consistent with the results of a previous study.^21^

The MB adsorption capacity is considered to be a function of adsorption time using the CNS carbons activated for various times and it is shown in Fig. 3B. There was a gradual adsorption in the first 80 min and then the adsorption ability reached its equilibrium value. When the activation time was increased, the CNS-based carbons activated within 30 min had a significantly lower adsorption capacity in MB removal at any adsorption time. This was compared to two other samples activated for 50 and 70 min.

There are two regimes in the dye adsorption profile: an external and an internal process^22^ that are expressed by a fast and a slow adsorption rate, respectively. The smooth and continuous adsorption curves in Fig. 4B show a low adsorption rate with the external adsorption dominating^23^ for all of the tested adsorbents. The aggregation of the MB molecules on the surface of the CNS-based activated carbons increased more and more, with the increase of adsorption time, forming an outside porous coverage and causing a new mass transport resistance for deeper MB diffusion.^23^ Therefore, formation of porous structures in the central core of the CNS-based activated carbons did not contribute any improvement to the MB adsorption with activation times up to 70 min.

### Determination of the adsorption mechanism

#### Empirical principle of advantage

The complex interactions among the MB molecules and the available adsorption sites are discussed in the paper of Khang *et al.* 2020.^24^ The adsorption rate was controlled by a one-step mechanism, which was determined by the underlying reaction mechanism in low MB concentration. At a high MB concentration, the adsorption processes were beyond the one-step mechanism, and a multi-step elementary reaction mechanism was considered. These complex reactions include: lateral interactions, multiple-binding sites and/or non-random adsorbate contribution. In recent times, the multi-step adsorption has been believed to contribute to the determination of the adsorption rate.^25,26^ One of the most important objectives in the study was to gain insight into and evaluate the adsorption mechanism of the MB molecules on the CNS-based activated carbon structures more comprehensively.

**Fig. 6:**
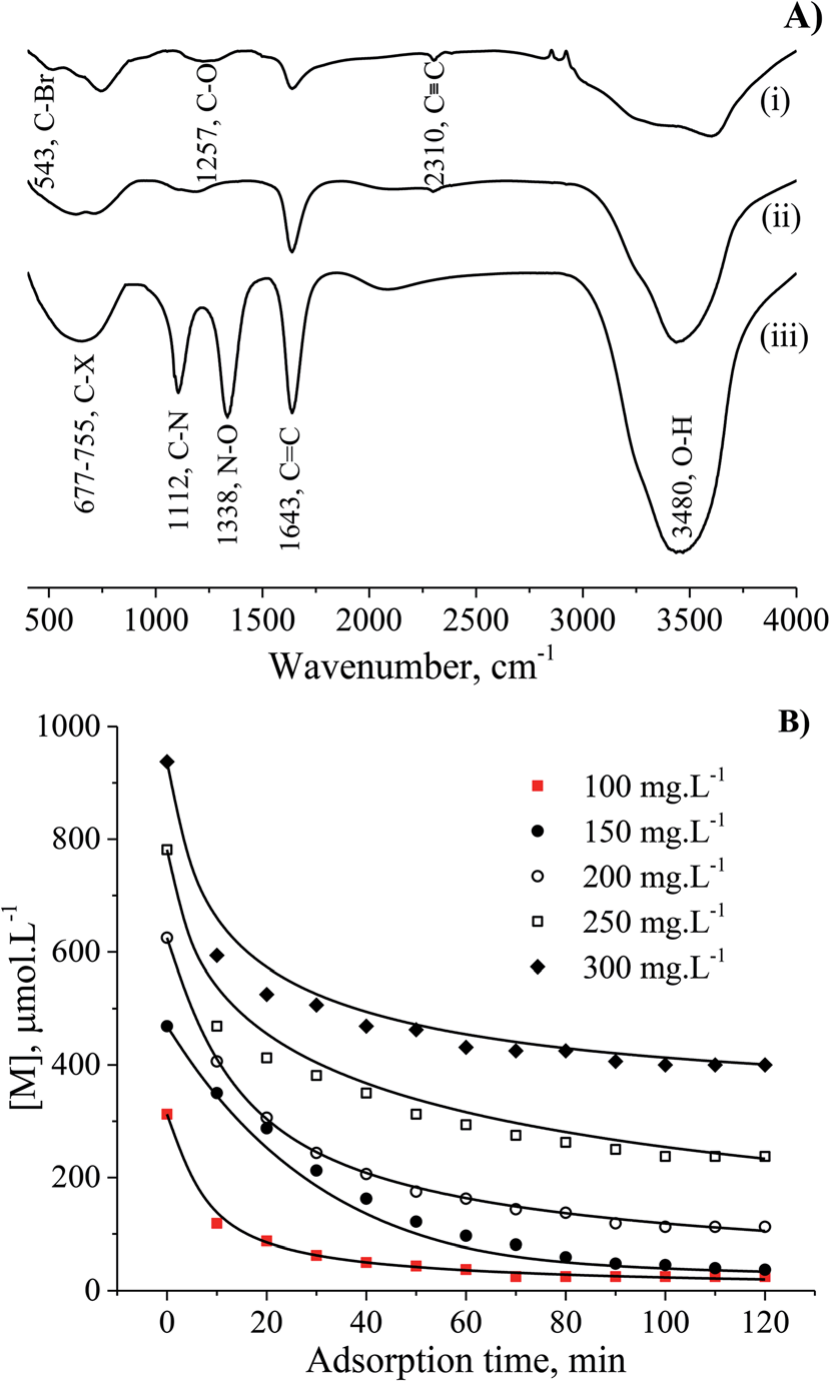
The vibrations of the chemical functional groups are shown in (A), (i) the carbonised CNS carbon, (ii) the CNS-based activated carbon before and (iii) after adsorption using FT-IR. (B) The adsorption kinetic curves of CNS-based activated carbon.

The interaction of MB molecules and the vibrations of chemical functional groups were detected by FT-IR analysis of the CNS-based activated carbons before and after adsorption tests as shown in Fig. 6A. The FT-IR spectrum of CNS carbonised (i) and CNS activated (ii) carbons showed the peaks of C–O, C=C, C≡C and O–H bonds at wavenumbers of 1257, 1643, 2310 and 3480 cm^−1^, respectively. The band at 543 cm^−1^ was assigned to C–“Br”, and the broad band from 677 to 755 cm^−1^ was attributed to C–X (X: F, Cl and/or I) stretching due to the presence of halogen atoms in materials^27,28^ and MB. It should be noted that the FT-IR curve (iii) for the utilised adsorbent with the appearance of high sharp peak at wavenumber of 1338 cm^−1^ is ascribed to the N–O bond which was absent in the FT-IR spectrum of MB in a previous report.^29^ In fact, the peak at a wavenumber of 1112 cm^−1^ in spectrum (iii) relates to the C–N stretching from the adsorbed MB molecules.

A variation of the constant *a* from the Elovich equation fit suggested that there was more than one-step governing the uptake of the MB adsorption.^29,30^ The complex adsorption processes were suggested to be due to: (1) deep diffusion of the adsorbate into the porous structure of the adsorbent, and (2) the liquid/solid interactions. It was difficult for the MB molecule to enter into the internal pores of the CNS-based activated carbon because of the larger sized MB molecules from 5.91 to 13.82 Å, compared to the pore sizes. Consequently, the external diffusion was not the rate-limiting step of the adsorption processes at any initial MB concentration. In general, both the internal and external diffusion, which approximated to item (1) above, were unable to explain the MB adsorption mechanism. The heterogeneous reactions of negatively charged groups (contributed by pH) in MB with the CNS activated carbon surface were in a complex liquid/solid interaction item (2) above.^31^

#### Multi-step reaction mechanism

The existence of the N–O bond on the adsorbent surface proved that the adsorbed MB molecule formed a chemical bond stretching with the active site either directly or indirectly. The MB adsorption was not affected by adsorption of water molecules as concluded in the previous section. However, the negatively charged adsorption sites were supported by the water solvent through a multi-step reaction mechanism. Therefore, it was predicted that the OH^−^ groups on the CNS-based activated carbon surfaces were reversibly ionised groups and become free active radicals at a pH of 9. The MB molecules were then trapped by these radicals by the N^*δ*+^ atoms in the MB structure, thus, generating the strong N–O bond. The elementary reactions are detailed in eqn (2) and (3):2
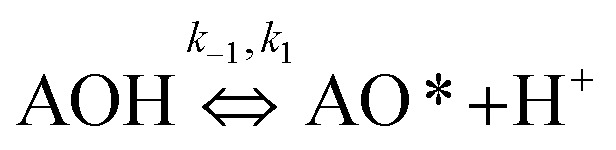
3
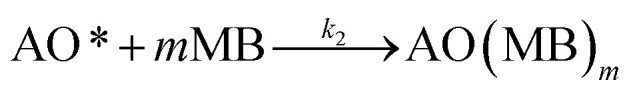
where AOH is an adsorbent surface including OH^−^ groups.

The adsorption step is descripted by eqn (3) and that conformed to adsorption kinetics in eqn (4):4
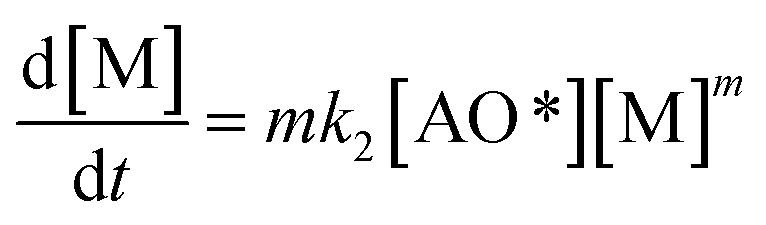


It is noted that the order of the adsorption kinetics is indicated by the value of *n* = *m* + 1, where, [M] is the relative concentration that indicates the mass dye concentration per unit of adsorbent in (mg L^−1^) g^−1^.

Applying the steady state approximation (SSA) approach for the predicted mechanism, the expression of the adsorption rate was established and is shown in eqn (5):5
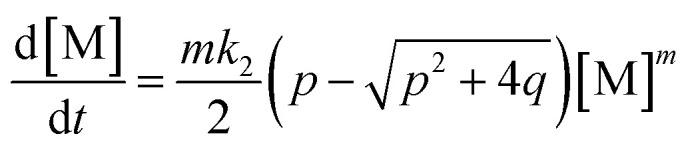
where, *p*, *q* related to [M] as functions:6
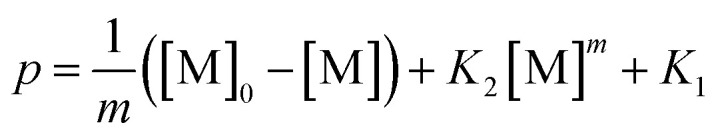
7
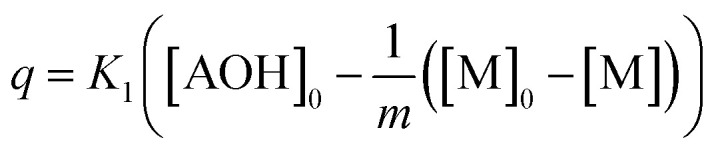
and 
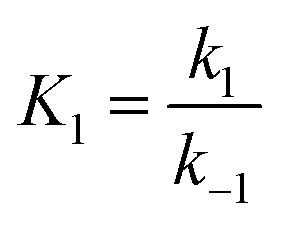
, 
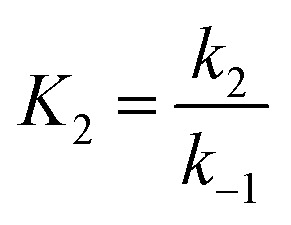
.

This differential eqn (5) was numerically solved and optimised in order to determine the *α*, *K*_1_, *K*_2_ and *k*_2_ parameters using the Runge–Kutta 4th method with a step height *h* = 1 min. The available adsorption sites on the adsorbent surface at is indicated by [AOH]_0_.

#### Validity of the established kinetic model

The study of Khang *et al.*^24^ showed that the experimental data were fitted well to the pseudo-first-order and the pseudo-second-order as well as the Elovich model. The established kinetic model (eqn (5)) was applied to fit the experimental adsorption kinetic data in this research. The results are summarised and shown in Table 2. The experimental data for the cation dye (Congo red and MB) adsorptions are described by the proposed model with a high correlation (*R*^2^ > 0.995). The applicability of eqn (5) for dye adsorption kinetic modelling was more significant for adsorption performed at high pH and with an initial dye concentration. The Elovich equation shows the best-fit-kinetic model to describe the MB adsorption at a high initial concentration. Nevertheless, the experimental data obtained from Ghaedi's publication^32^ exhibited a more suitable model compared to the proposed model due to the higher *R*^2^.

**Table 2:** The calculated parameters from the established kinetic model fits^a^

Ref., adsorbate/adsorbent, pH	[M]_0_, (mg L^−1^) g^−1^	Best-fit-kinetic model in the reference		Calculated parameters				
		Model	*R* ^2^	*p*	*K*_1_	*K*_2_	*k*_2_	*R* ^2^
10, Congo red/CNS, pH = 3	1	Pseudo-second-order	0.999	1.47	0.17	290.8	1.57 × 10^−3^	0.9967
	3		0.999	2.02	2.54 × 10^−4^	1.5	0.049	0.9974
	5		0.999	2.55	6.84 × 10^−5^	0.4	0.167	0.9963
10, Congo red/CNS, pH = 10	1	Pseudo-second-order	0.998	0.96	4.73 × 10^−5^	0	2.41	0.9993
	3		0.998	1.36	0.12	3.96 × 10^−4^	4.26 × 10^−2^	0.9976
	5		0.996	1.63	3.22	9.71 × 10^−3^	2.96 × 10^−4^	0.9967
32, MB/AC from peanut stick, pH = 5	3.89	Elovich	0.9986	0.16	0.532	0	3.713	0.9995
	7.22		0.9972	1.05	0.133	10^−4^	0.712	0.9997
	11.1		0.9968	0.86	1.42 × 10^−4^	1.36 × 10^−5^	5.911	0.9994
This work, MB/CNS, pH = 9	100			2.18	0.015	0	4.42 × 10^−6^	0.9979
	150			1.14	0.434	3.028	4.06 × 10^−6^	0.9978
	200			2.37	0.023	1.42 × 10^−3^	5.12 × 10^−6^	0.9991
	250			2.67	0.001	5.24 × 10^−6^	1.68 × 10^−4^	0.9981
	300			2.36	0.141	2.09 × 10^−2^	3.90 × 10^−3^	0.9951

a Abbreviations: MB – methylene blue, AC – activated carbon, CNS – cashew nut shells.

The overall observations of *K*_1_ and *K*_2_, showed the low values (less than 1). This indicated that using eqn (2) the rate of the forward reaction was less than that the reverse reaction. As a result of this, the AO* active radicals and the available adsorption sites existed in an unstable state and it was easy for them to re-combined with an H atom. Therefore, the dye molecule must immediately hit the just generated AO* in order to form adsorptive stretching. In addition, the dye molecules are known to have a bulky structure which reduced the probability of the dye molecule being attracted to the AO* radical because of the low values of rate constant *k*_2_. The rates of the elementary reactions in equations of (2) and (3) contributed to the understanding of the adsorption mechanism and adsorption kinetics, which depended on several factors including temperature, stirring speed and properties of the solution.

The feasible agreement of the proposed model with the experimental data for all the [M]_0_ values was indicated by the closeness of *R*_2_ to unity as shown in Table 2 and the fitting curves in Fig. 6B. The factor of the adsorbed adsorbate number on an active site was evaluated from the adsorption kinetic order (*m* + 1) with changes from 2.14 to 2.67, and the mean order was found at 3.14 using various initial concentrations. Moreover, the number of MB layers on an adsorbent surface was determined from the n-layer BET isotherm model fitting with a resultant value of 4.63.

## Conclusions

The cashew nut shell-based activated carbon preparation and the mechanism of MB absorption on this product were investigated and discussed. The results show that the specific surface area and absorption capacity of the CNS-based activated carbon were improved significantly in comparison with the results of previous studies on the CNS-based activated carbon.^30^ The products obtained also prove that the waste from cashew nut production has the potential to be utilised as a raw material for producing high quality activated carbon and which could be used as a commercialised product in waste water treatment. Methylene blue was used as an absorbate to comprehensively investigate the adsorption mechanism of the CNS-based activated carbon material. The regression models of the MB adsorption were used to optimise the operating conditions when the CNS-based activated carbon was applied to remove pollutants from waste water. The original findings in this study could be the basis for future studies on other effects such as flow regime, particle size, retention time, and so on.

## Conflicts of interest

There are no conflicts to declare.

## Supplementary Material

RA-011-D1RA04672A-s001
